# Case Report: Safety and Efficacy of Tocilizumab in a Patient with Rheumatoid Arthritis and Chronic Hepatitis C

**DOI:** 10.1155/2012/212381

**Published:** 2012-02-16

**Authors:** Filippo Iebba, Fiorella Di Sora, Agapito Tarasi, Wilma Leti, Tatiana Montella, Francesco Montella

**Affiliations:** Division of Clinical Immunology, San Giovanni-Addolorata Hospital, 00184 Rome, Italy

## Abstract

Tocilizumab is a monoclonal humanized anti-IL-6-receptor antibody used for the treatment of rheumatoid arthritis. The safety of tocilizumab in HCV patients is an open question. We report on safety and efficacy of tocilizumab in a 71-year-old female with rheumatoid arthritis and chronic hepatitis C. Monotherapy with tocilizumab (8 mg/kg every 4 weeks, i.v.) was prescribed after the discontinuation, determined by clinical inefficacy, of anti-TNF-alfa agents (adalimumab and, subsequently, etanercept). We have registered an optimal and rapid clinical response to tocilizumab with early remission (SDAI <3.3 since 4 weeks). The safety was good with no adverse events and maintenance, during a six-month followup, of normal liver enzymes. These data suggest a good safety profile of tocilizumab in patients with rheumatoid arthritis and chronic hepatitis C virus pathology.

## 1. Introduction

Tocilizumab (TCZ) is a monoclonal humanized anti-Interleukin-6-(IL-6-) receptor antibody, which has been shown to be highly and rapidly efficacious for the clinical and radiographic outcome of patients with severe rheumatoid arthritis (RA) [1]. TCZ is efficacious also as a monotherapy, is capable to induce a rapid remission of arthritis activity, and ameliorates the systemic manifestations of rheumatoid arthritis. The safety profile of tocilizumab in clinical trials was good with adverse events rate similar to control groups. In “real life,” the safety profile of tocilizumab in RA patients with chronic hepatitis C is an open question. Actually (on 02 December 2011) there is only one article in PubMed, a case report [2]. Some studies have evidenced a potential pathogenetic role of IL-6 for the liver and systemic manifestations of HCV infection [3, 4]. This evidence permits to hypothesize an interference of tocilizumab on the course of HCV pathology. In this paper we have described the efficacy and safety profile of tocilizumab during a 24-week followup of an HCV patient with severe rheumatoid arthritis, nonresponder to methotrexate and TNF-alfa agents.

## 2. Case Report

A 71-year-old Caucasian female patient presented to our division on 18 April 2011. She had been diagnosed with chronic hepatitis C (at 51 year of age) and rheumatoid arthritis (at 58 year of age). Hepatitis C virus genotype was 2a/2c. A liver biopsy, at 57 year of age, documented a chronic active hepatitis of moderate severity with inflammatory intralobular and portal infiltrates, and moderate piece-meal necrosis. The clinical course of chronic HCV pathology was benign with constant normality of alanine/aspartate aminotransferase (ALT/AST), and absence of liver or systemic impairment. Ultrasonography documented an augment of liver volume, a homogeneous echostructure, and the absence of focal lesions or cirrhosis. Rheumatoid arthritis was severe since onset and was immediately treated with methotrexate (15–20 mg every 7 days). At 67 year of age, the patient began an anti-TNF-alfa treatment with adalimumab (40 mg s.c. every 14 days) in combination with methotrexate (20 mg i.m. every 7 days) with rapid and sustained response (remission) up to 70 years of age when there was a strong relapse of RA. Methotrexate was interrupted for individual gastrointestinal intolerance. At October 2010, the patient started monotherapy with etanercept (50 mg s.c. every 7 days) without a significant improvement of disease activity (after six months, SDAI ( simplified disease activity score ) >40). At the first visit at our division (on 18 April 2011), she has shown the persistence of an elevated activity of rheumatoid arthritis (SDAI = 50,5) with numerous tender and swollen joints, prolonged (some hours) morning stiffness and strong disability. The analysis of liver exams showed normal values of all parameters. The result of quantitative HCV-RNA was 6962286 IU/mL. The levels of alfa-fetoprotein were normal (2,9 ng/mL, normal values <7). Anticyclic citrullinated peptide antibodies (anti-CCP) were positive. Rheumatoid factor was negative. We have prescribed the interruption, for inefficacy, of treatment with etanercept. On 3 June 2011, the patient began monotherapy with tocilizumab (8 mg/kg i.v. every four weeks). At this date (week 0) and during followup (weeks 2, 4, 8, 12, 16, 20, 24), we have determined and collected the subsequent parameters:

- *liver exams*: alanine aminotransferase (ALT, U/L, normal <31), aspartate aminotransferase (AST, U/L, normal <31), gamma-glutamyl transpeptidase (GGT, U/L, normal <32), alkaline phosphatase (ALP, U/L, normal <360), total bilirubin (mg/dL, normal <1,1), direct bilirubin (mg/dL, normal <0,5);
- etoricoxib (90 mg) requirement (number of tablets/week);
- *SDAI* (Simplified Disease Activity Index): it is a validated index of activity of rheumatoid arthritis. SDAI is the numerical sum of 5 parameters: tender joint count (0–28), swollen joint count (0–28), patient global assessment of disease activity (0–10), physician global assessment of disease activity (0–10), and level of C-reactive protein (mg/dL). An SDAI value of >40 indicates high activity of disease, an SDAI value of 20–40 indicates a moderate RA activity, an SDAI value <20 indicates a mild disease, an SDAI value <3.3 indicates the remission of rheumatoid arthritis.

The monotherapy with tocilizumab, in this patient, has determined a rapid clinical improvement: after 2 weeks, we have verified a significant reduction of SDAI, from 59,24 to 4. The remission of rheumatoid arthritis (SDAI <3,3) was already registered after 4 weeks of treatment and persisted during all the followup (at present 24 weeks).

**Figure 1:**
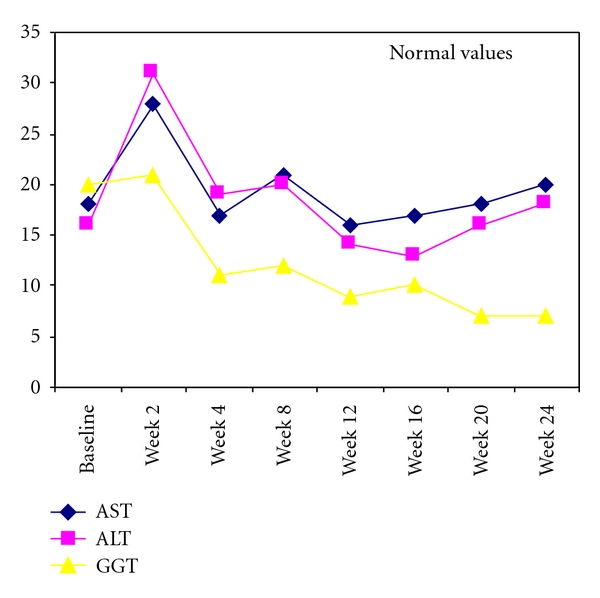
Levels of liver enzymes during treatment with tocilizumab.

**Figure 2:**
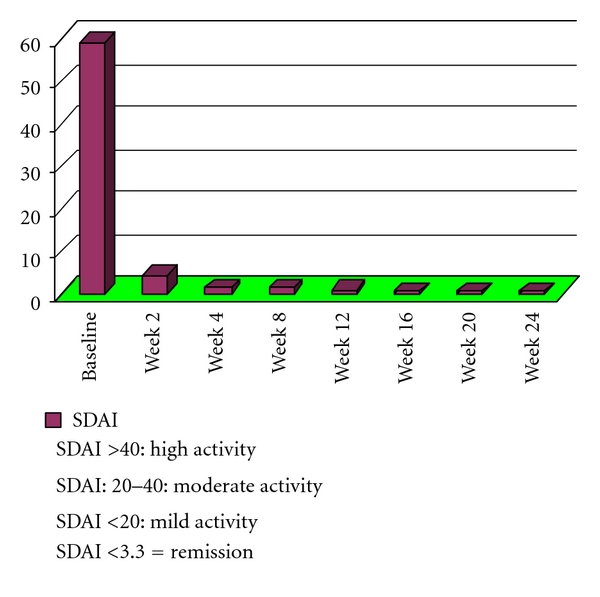
Rheumatoid arthritis activity (SDAI) during treatment with tocilizumab.

The analysis of liver exams has shown the maintenance of normal liver function during all the followup. No signs of cirrhosis were detected. No other adverse events associated to tocilizumab treatment were registered during the observation period. The etoricoxib requirement, high at baseline (5 tablets/week), was reduced to 1 tablet/week after 2 weeks and to 0 tablet/week after 4 weeks. The results of this study were collected in Table 1. The course of AST, ALT, and GGT was shown in Figure 1. The evaluation of activity of rheumatoid arthritis with SDAI during the treatment with tocilizumab was illustrated in Figure 2.

## 3. Discussion

Biologic therapy (anti-TNF-alfa agents, abatacept, rituximab, tocilizumab) has dramatically improved the clinical and anatomic course of rheumatoid arthritis. The use of these agents in patient with early arthritis can determine a rapid clinical remission and an arrest or, at least, a substantial slowing of radiological progression. Tocilizumab, particularly, has shown a high and rapid efficacy and is the sole biologic agent that has been superior to methotrexate as monotherapy for the clinical improvement of rheumatoid arthritis. The safety profile of tocilizumab showed by clinical trials is good. The most common adverse events associated to tocilizumab treatment were infections, gastrointestinal symptoms, alteration of liver enzymes, hypercholesterolemia, neutropenia, and infusion reactions. The rate of adverse events was similar between tocilizumab and control groups. Most adverse events associated to tocilizumab were mild or moderate. The tolerability and safety of tocilizumab in RA patients with HCV infection in clinical practice is a relevant open question. An analysis of medical literature with PubMed (keywords: tocilizumab and Hepatitis C Virus), executed on 02 December 2011, has documented the presence of only one study, a case report by Nagashima and coworkers. Serum IL-6 and its soluble receptor levels were correlated with liver function impairment and liver fibrosis. In HCV patients with chronic hepatitis, cirrhosis, hepatocellular carcinoma, or HCV-related mixed cryoglobulinemia, augmented serum levels of IL-6 were detected [5, 6]. The potential pathogenetic contribute of IL-6 for the development of liver and systemic manifestations of HCV infection suggests a possible interference of tocilizumab on outcome of chronic Hepatitis C. Our paper has shown the absence of liver impairment of tocilizumab with maintenance of normal AST, ALT, GGT, ALP, and bilirubine levels during all the followup. These results suggest, as well as for anti-TNF-alfa agents, a good liver safety of tocilizumab in RA patients with HCV infection. These suggestions obviously have to be confirmed by controlled and randomized trials and/or large observational studies. The rapid remission of arthritis determined in our patient by tocilizumab is in complete accordance with the trial results and is a crucial objective of biologic therapy, particularly in patients with early arthritis, because recent studies show that the rapidity of remission is strictly associated to a better radiologic outcome of rheumatoid arthritis. The rapid interruption, since 4 weeks, of etoricoxib intake was another positive effect of tocilizumab on the course of rheumatoid arthritis and HCV disease in this patient.

## Figures and Tables

**Table 1:** Liver function, etoricoxib requirement, and arthritis activity at baseline and during treatment with tocilizumab in a 71-year-old female patient with rheumatoid arthritis and HCV Infection.

Parameter	Week 0	Week 2	Week 4	Week 8	Week 12	Week 16	Week 20	Week 24
AST (U/L, normal values <31)	18	28	17	21	16	17	18	20
ALT (U/L, normal values <31)	16	31	19	20	14	13	16	18
GGT (U/L, normal values <32)	20	21	11	12	9	10	7	7
ALP (U/L, normal values <360)	136	155	121	120	133	124	122	139
Total bilirubine (mg/dl, normal values <1.1)	0,61	0,70	0,78	0,70	0,91	0,66	0,56	0,80
Direct bilirubine (mg/dl, normal values <0.5)	0,15	0,18	0,20	0,19	0,23	0,19	0,14	0,21
Etoricoxib 90 mg tablet requirement (numbers/week)	5	1	0	0	0	0	0	0
SDAI (remission <3,3)	59,24	4	1	1	0,5	0,1	0,2	0,2
